# RETRACTION: A Meta‐Analysis Comparing the Effects of Cemented and Uncemented Prostheses on Wound Infection and Pain in Patients With Femoral Neck Fractures

**DOI:** 10.1111/iwj.70311

**Published:** 2025-03-06

## Abstract

**RETRACTION**: XuZ. , ZhangK. , ChengK. , SunG. , ZhangY. , and JiaJ. , “A Meta‐Analysis Comparing the Effects of Cemented and Uncemented Prostheses on Wound Infection and Pain in Patients With Femoral Neck Fractures,” International Wound Journal 20, no. 10 (2023): 4122–4129, 10.1111/iwj.14306.37555547 PMC10681411

The above article, published online on 09 August 2023, in Wiley Online Library (http://onlinelibrary.wiley.com/), has been retracted by agreement between the journal Editor in Chief, Professor Keith Harding; and John Wiley & Sons Ltd. Following an investigation by the publisher, all parties have concluded that this article was accepted solely on the basis of a compromised peer review process. The editors have therefore decided to retract the article. The authors did not respond to our notice regarding the retraction.

**RETRACTION**: Z. Xu , K. Zhang , K. Cheng , G. Sun , Y. Zhang , and J. Jia , “A Meta‐Analysis Comparing the Effects of Cemented and Uncemented Prostheses on Wound Infection and Pain in Patients With Femoral Neck Fractures,” International Wound Journal 20, no. 10 (2023): 4122–4129, 10.1111/iwj.14306.37555547 PMC10681411

The above article, published online on 09 August 2023, in Wiley Online Library (http://onlinelibrary.wiley.com/), has been retracted by agreement between the journal Editor in Chief, Professor Keith Harding; and John Wiley & Sons Ltd. Following an investigation by the publisher, all parties have concluded that this article was accepted solely on the basis of a compromised peer review process. The editors have therefore decided to retract the article. The authors did not respond to our notice regarding the retraction.

